# Gender Role Characteristics and Entrepreneurial Self-Efficacy: A Comparative Study of Female and Male Entrepreneurs in China

**DOI:** 10.3389/fpsyg.2020.585803

**Published:** 2020-12-17

**Authors:** Chengyan Li, Diana Bilimoria, Yelin Wang, Xiaowei Guo

**Affiliations:** ^1^Department of HRM, Shanghai Normal University, Shanghai, China; ^2^Department of Organizational Behavior, Case Western Reserve University, Cleveland, OH, United States; ^3^Department of Education and Human Development, New York University, New York, NY, United States; ^4^Shanghai University of International Business and Economics, Shanghai, China

**Keywords:** gender role orientation, entrepreneurial self-efficacy, female entrepreneur, male entrepreneur, China

## Abstract

This study, based on Bem’s (1974) gender schema theory, investigates gender differences in and the relationship between gender role characteristics and entrepreneurial self-efficacy (ESE) of 261 female and 265 male entrepreneurs in China. The results show that male and female entrepreneurs did not differ significantly in ESE or in masculine gender role characteristics, but differed significantly in feminine gender role characteristics. Examining four different stages in the entrepreneurial life cycle, we find that for female entrepreneurs, feminine characteristics had a positive influence on ESE in the searching and planning stages of entrepreneurship, and masculine characteristics had a positive influence on ESE in the searching stage. For male entrepreneurs, feminine characteristics had a positive influence on ESE in the searching and planning stages, and masculine characteristics had a positive influence on ESE in the marshaling and implementing stages. In addition, one feminine characteristic, “Friendly,” showed a positive association with male entrepreneurs’ ESE in the marshaling stage. Overall, the feminine gender role factor of “Friendly” and the masculine gender role factor of “Compete” played a greater role on ESE than other characteristics. Implications of the findings are discussed. This study contributes a new perspective to extant research on entrepreneurial self-efficacy and female entrepreneurship.

## Introduction

Previous research offers diverse views of the relationship between gender and entrepreneurial self-efficacy (ESE). Women embark on entrepreneurial careers less often than men do, which some studies contend may be ascribed to a higher level of ESE of male entrepreneurs than female entrepreneurs. For example, Scherer et al. (1990) found that the ESE of female MBA students was lower than that of male MBA students, and that female students were also lower than male students in their aspirations of entrepreneurship as a career choice. Wilson et al. (2007) found that gender differences in ESE appear early, with implications for entrepreneurial career choices. Other scholars contend that women appear to be less self-assured that they have the skills of starting up companies (Koellinger et al., 2008). Yet, Chen et al. (1998) and Zhao et al. (2005) found that gender is not associated with ESE. Building on previous scholarship on the relationship between gender and ESE, the present study examines gender differences in and the relationship between gender role characteristics and ESE in a Chinese context.

Entrepreneurial self-efficacy is an important psychological variable in entrepreneurship. Most previous studies have focused on the impact of ESE on entrepreneurial intention, entrepreneurial behavior and entrepreneurial performance, representing the formative mechanism of ESE on various entrepreneurial outcomes. As Palmer et al. (2019) note, few studies examine ESE from the perspective of the psychological characteristics of senior leaders of SMEs. By focusing on the gender role characteristics of entrepreneurs, the present study addresses this gap.

A notable example of the study of entrepreneurs’ psychological characteristics is Stephen and Mary (2008), who examined the influence of entrepreneurs’ gender-role identification on ESE, and found a positive association. However, their study was based on MBA students (only 11% of the 216 individuals studied had their own companies). The applicability of their conclusions to entrepreneurs needs to be examined and verified. Based on this previous study by Stephen and Mary (2008), in the present study we examined the impact of gender-role identification on ESE for 526 Chinese entrepreneurs (265 males and 261 females). Our study further subdivides the structure of gender roles into masculine and feminine dimensions, discusses the impact of each dimension on ESE at each of the four stages of entrepreneurship: searching, planning, marshaling, and implementing (Wilson et al., 2007; Kickul et al., 2009), and compares the relationship between gender role orientations and ESE for male and female entrepreneurs. The main research questions of the study are: (1) Is there a gender difference in ESE? and (2) What are the effects of gender role orientations on ESE and is there a gender difference in the effect?

## Theory and Hypotheses

### Gender Role Orientation

Gender role theory posits that gender roles consist of people’s expectations and beliefs about normative gender differences in psychological and behavioral characteristics (Eagly et al., 2008). Gender roles are social roles that encompass behaviors and attitudes considered acceptable or appropriate based on a person’s biological or perceived sex. Constantinople and Anne (1973) developed a theoretical framework, in which the similarity of the two genders were discussed. In this framework, Constantinople (1973) proposed that males and females are two separated structures, rather than the two poles of a continuum. Building on this perspective, Bem (1974) created the Bem Sex Role Inventory (BSRI) which recognizes that individuals may demonstrate both masculine and feminine characteristics. Bem’s views were widely applied in later research (e.g., Spence et al., 1980; Stieger et al., 2014). Studies on the influence of socialization on male and female college students’ gender roles indicate that men’s masculinity is considerably higher than women’s, and women’s femininity is higher than men’s (Stephen and Mary, 2008; Zhi, 2011).

Previous research indicates that all males, including those that exhibit masculine and feminine characteristics, are attributed to possess the psychological characteristics (i.e., the self-concept and self-esteem) suitable for being a leader (Kent and Moss, 1994; McCabe et al., 2006). Drydakis et al. (2018) found that women who exhibit masculine personality traits are more competitive than those displaying feminine personality traits.

In the entrepreneurship literature, Holm et al. (2013) found that a sample of Chinese entrepreneurs had a higher desire to enter competition than non-entrepreneurs, which is consistent with male gender role characteristics. While Gneezy et al. (2003) found that males performed better than females in competitive environments, a study of 105 female entrepreneurs and 69 male entrepreneurs found that female entrepreneurs have a higher demand for autonomy and the experience of new things (Sexton and Bowman-Upton, 1990), which are also masculine gender role traits. Masculine personality traits increase perceptions of competency levels and leadership capability, and therefore both male and female entrepreneurs benefit from exhibiting masculine characteristics. Further, since the male and female respondents in the present study are established entrepreneurs, both groups are likely to be confident (a masculine role trait) in their entrepreneurial abilities.

Since feminine characteristics are not as highly valued as masculine characteristics for leadership, and since women are more likely to exhibit feminine characteristics, we hypothesize a gender difference between male and female entrepreneurs in feminine gender role traits, but not masculine gender role traits. Thus,

Hypothesis 1. There are no significant differences between male entrepreneurs and female entrepreneurs in masculine role characteristics, but there are significant differences in feminine role characteristics.

### Entrepreneurial Self-Efficacy

Self-efficacy is a core concept influencing an individual’s motivation and behavior (Albert, 1990; Bandura, 1990). It is “the perception of one’s capabilities to attain performance outcomes” (Audia et al., 2000, p. 4). Related to entrepreneurship, self-efficacy can drive an individual to conquer the various hurdles and challenges of starting-up and operating an enterprise (Shanea et al., 2003). One of the central ideas in self-efficacy theory is that engagement and persistence in a given activity is a function of an individual’s assessment of their skills and capabilities to successfully accomplish the activity as well as cope with challenges in the environment (Arshad et al., 2020).

Entrepreneurial self-efficacy (ESE) is the specific application of Bandura’s (1990) concept of self-efficacy to entrepreneurship (Chen et al., 1998). Chen et al. (1998) proposed that ESE refers to an individual’s confidence in achieving the role of an entrepreneur and completing entrepreneurial tasks, and suggested that it is one of the predictive variables of the likelihood of being an entrepreneur. ESE reflects the ability to prevent and control negative actions and thoughts (Drnovsek et al., 2010). Previous research has found that ESE is positively related to firm performance (e.g., Miao et al., 2017).

ESE develops from entrepreneurial socialization as entrepreneurs deal with various unexpected problems and difficulties in the process of establishing a business. Since they have successfully started a business, ESE is generally higher for entrepreneurs than the general public (van der Westhuizen and Goyayi, 2020). While male and female entrepreneurs should have higher ESE levels than the general population, there is no theoretical reason to hypothesize an innate gender difference in ESE. Since our study investigates those who have already established entrepreneurial businesses, we posit that there will be no differences in ESE between male and female entrepreneurs.

Although there are fewer female entrepreneurs than male entrepreneurs, we posit that women are less likely to intend to or become entrepreneurs due to gender-restrictive social norms, and not because women’s ESE is lower than men’s. Attitudes toward entrepreneurship, which have a positive impact on entrepreneurial intentions, are driven by social norms. Social norms regulate individuals’ attitudes and lead to the adoption of socially acceptable actions. Arshad et al. (2016) found that while ESE had a greater effect on the entrepreneurship attitudes of males than females, perceived social norms had a greater effect on female entrepreneurship attitudes. Entrepreneurship attitudes of women were found to be primarily driven by community feelings and aspirations (Arshad et al., 2020). Social norms regarding gender roles generally hold that men are more suitable as entrepreneurs since they are perceived as more agentic, independent, and as working outside the home (Delmar and Davidsson, 2000; Eagly et al., 2008) and women are perceived as more communal and more concerned with the harmonious functioning of groups and interrelationships (Skitka and Maslach, 1996; Eagly et al., 2008). Thus, we hold that gender difference in intentions to become an entrepreneur is not caused by gender differences in ESE, but the result of social norms. In previous research, entrepreneurship has been found to enhance women’s empowerment, self-drive, and autonomy (Zgheib, 2018).

Hypothesis 2. There is no difference in the ESE of male and female entrepreneurs.

### The Relationship of Gender Roles and ESE

To better understand the relationship between male and female entrepreneurs’ gender role characteristics and their ESE, this study investigates ESE at four stages of entrepreneurship: searching stage, planning stage, marshaling stage, and implementing stage (Wilson et al., 2007; Kickul et al., 2009). Each stage of the entrepreneurial life cycle has multiple functions, with 10 different tasks (Wilson et al., 2007).

The effect of gender role identification on entrepreneurial self-efficacy is first reflected in entrepreneurial opportunity identification. Because of their different cognitive styles, male and female entrepreneurs may differ in their recognition of entrepreneurial opportunities. Entrepreneurial opportunity identification is an information processing practice that depends on individual cognitive characteristics (Gaglio and Katz, 2001). Cognitive style can influence the preferences of individual entrepreneurial cognition. Individuals with an intuitive cognitive style tend to pursue unique ideas rather than sticking to rules and standards. They are more creative and have higher innovation performance than individuals with an analytic cognitive style (Kim et al., 2012). Individuals with an intuitive cognitive style are better at identifying entrepreneurial opportunities, and they are more likely to identify opportunities for innovation (Baldacchino, 2013).

When entrepreneurs focus on identifying entrepreneurial opportunities (trying to develop a new product, service, or technology), they tend to think intuitively. Intuition reflects differences among individuals based on environmental event sensitivity and is a key element in identifying opportunities for entrepreneurs (Kozhevnikov et al., 2014). Intuition is an unconscious, holistic, fast, emotion-driven judgment process (Dane and Pratt, 2007; Kozhevnikov et al., 2014). In the process of their socialization, women learn the skills of relationship and empathy, and are socialized to be sensitive to their environments and think in intuitive ways. Entrepreneurs with more feminine characteristics tend to employ an intuitive cognitive style, which is more effective in identifying entrepreneurial opportunities.

Identifying entrepreneurial opportunities can stimulate entrepreneurs’ confidence and motivation, which helps to enhance their self-efficacy in the stage of entrepreneurial search. Therefore, we expect that:

Hypothesis 3. Feminine gender role characteristics are positively associated with the ESE of male and female entrepreneurs in the searching stage.

The cognitive style of business leaders has an impact on entrepreneurship and is related to innovation ability (Li et al., 2020). Jeffrey et al. (2012) suggested that an individual is more likely to be an entrepreneur if they are aggressive, adventurous and self-disciplined. These characteristics are typically regarded as masculine gender role characteristics. Individuals with masculine gender role characteristics tend to be rational and have an analytical cognitive style. An entrepreneur’s rational, analytic, and cause-and-effect-oriented processes organizes their intent and action, and are the foundation of a formal business plan, opportunity analysis, resource acquisition, goal setting, and observable goal-directed behavior (Bird, 1988). Olson (1985) also pointed out that when entrepreneurs plan and execute new enterprises, their information processing is significantly analytical. Brigham et al. (2007) showed that individuals with an analytic cognitive style show higher self-efficacy than those with an intuitive cognitive style, and they tend to get more satisfaction during the planning stage.

Similar to the planning stage, the marshaling or resource integration stage also requires analysis and processing, especially when financing business endeavors. A CEO’s cognitive ability helps them to make decisions that are conducive to maintaining the sustainable development of the company (Sarfraz et al., 2020). The marshaling stage also demands high competitiveness, enterprise and networking capabilities, especially in raising necessary resources and financing for the business. Since community culture plays a large role in ESE (Coleman and Kariv, 2014), masculine characteristics will likely enable enhanced access to community resources and financing.

In the implementing stage of entrepreneurship, individuals demonstrating male-typed analytic characteristics likely exhibit greater trust in their own abilities, and stronger confidence in accomplishing activities like prediction, preparing, designing, and organizing. These logics are aligned with Kickul et al. (2009) who suggested that individuals with an analytic cognitive style are likely to demonstrate stronger ESE at the planning, marshaling, and implementing stages of entrepreneurship than those with the intuitive cognitive style. Thus,

Hypothesis 4. Masculine gender role characteristics are positively associated with the ESE of male and female entrepreneurs in the planning, marshaling and implementing stages.

## Methods

Data were collected through an anonymous survey, conducted in accordance with the ethical rules and approval of the Ethics Committee of Shanghai Normal University, China. All participants gave informed consent.

### Sample

The study’s sample consisted of male and female entrepreneurs of small and medium sized businesses in the developed regions of East China. We selected this region because it is active in entrepreneurship, with many entrepreneurs in this region. We conducted a two-phase questionnaire survey. In the first-phase survey, we distributed questionnaires to female entrepreneurs studying in Executive MBA programs at institutions in which we teach as well as other entrepreneurs in our personal networks. In the second-phase survey, we turned to the head teachers of a primary school in Zhejiang Province of China for help in distributing questionnaires to their elementary school students’ fathers who are entrepreneurs. We distributed 350 questionnaires to female entrepreneurs and collected 312 completed survey responses. 370 questionnaires were distributed to male entrepreneurs and 325 completed survey responses were collected. After filtration, the final number of the usable questionnaires was 526, of which 261 were from female and 265 were from male entrepreneurs. Supplementary Appendix 1 provides the questionnaire items. Table 1 shows descriptive information about the sample.

**TABLE 1:** Sample description.

	Female (*N* = 261)			Male (*N* = 265)		
		
	Category	n	%	Category	n	%
Age	20–30 years old	63	24.1	20–30 years old	8	3.0
	31–40 years old	142	54.4	31–40 years old	147	55.5
	41–50 years old	43	16.5	41–50 years old	96	36.2
	Over 50 years old	11	4.2	Over 50 years old	13	4.9
	Missing value	2	0.8	Missing value	1	0.4
Enterprise creation time	Below 1 year	24	9.2	Below 1 year	13	4.9
	1–3 years	77	29.5	1–3 years	64	24.2
	4–5 years	40	15.3	4–5 years	57	21.5
	5–10 years	53	20.3	5–10 years	74	27.9
	Above 11 years	57	21.8	Above 11 years	55	20.8
	Missing value	10	3.8	Missing value	2	0.8
Industry	Manufactory	39	14.9	Manufactory	73	27.5
	Service/trade	129	49.4	Service/trade	86	32.5
	High technology	11	4.2	High technology	14	5.3
	Finance/real estate	13	5.0	Finance/real estate	17	6.4
	Others	59	22.6	Others	70	26.4
	Missing value	10	3.8	Missing value	5	1.9
Education	High school and below	84	32.2	High school and below	139	52.5
	Community college	87	33.3	Community college	63	23.8
	Bachelor degree and above	72	27.6	Bachelor degree and above	53	20.0
	Master degree and above	12	4.6	Master degree and above	8	3.0
	Missing value	6	2.3	Missing value	2	0.8
No. of employee	1–10	140	53.6	1–10	108	40.8
	11–50	64	24.5	11–50	80	30.2
	50–99	15	5.7	50–99	31	11.7
	100–199	15	5.7	100–199	27	10.2
	Above 500	14	5.4	Above 500	13	4.9
	Missing value	13	5.0	Missing value	6	2.3
Enterprise nature	Family business	22	8.4	Family business	21	7.9
	General private business	128	49.0	General private business	134	50.6
	Self-employed business	105	40.2	Self-employed business	108	40.8
	Missing value	6	2.3	Missing value	2	0.8

### Measures

We utilized Bem’s Sex Role Inventory (BSRI) to measure gender role characteristics. BSRI is one of the most widely used measures of gender roles (Szymanowicz and Furnham, 2013; Stieger et al., 2014), whose effectiveness has been established by various research studies and applications (Katsurada and Sugihara, 1999; Hoffman and Borders, 2001). The BSRI rating scale is formatted with 40 items, 20 in the masculine sub-scale, 20 in the neutral sub-scale and 20 in the feminine sub-scale. The neutral sub-scale was originally used to distinguish between masculine and feminine characteristics when the scale was compiled. Subsequent research using the BSRI has not employed the 20 items of the neutral sub-scale. Since the purpose of the present study is to focus on the influence of masculine and feminine gender role characteristics on ESE, we employ only the 40 items in the masculine and feminine sub-scales, and not the 20 additional items in the neutral sub-scale.

We conducted an exploratory factor analysis on half of the sample data (*n* = 262) and found that gender roles could be split into seven factors, with loadings of each item being over 0.5 (see Supplementary Appendix 2). The seven factors were: Self-government, Enterprise, Power, and Compete as four masculine gender role factors and Forthright and Sincere, Friendly, and Empathy as three feminine gender role factors. The four masculine factors were from the BSRI masculine sub-scale and the three feminine factors were from the BSRI feminine sub-scale. We used AMOS statistical software to conduct a confirmatory factor analysis on the other half of the sample data (*n* = 264) and the results indicated high construct validity (the Fit Index of the confirmatory factor analysis was: χ^2^ = 379.08; *df* = 250; χ^2^/*df* = 1.52; GFI = 0.90; AGFI = 0.87; CFI = 0.92; IFI = 0.92; PNFI = 0.67; RMSEA = 0.044). The reliability coefficient of the overall BSRI scale was 0.838, while the feminine and masculine sub-scales had reliability coefficients of 0.770 and 0.842, respectively.

To measure ESE, we used the scale developed by Kickul et al. (2009), including 10 task items and the four stages of entrepreneurship, searching, planning, marshaling, and implementing. Kickul et al. (2009) believed the ESE four-factor model has the best performance. The reliability coefficient of the ESE scale was 0.856. All statistical analyses in this study were conducted using SPSS 16.0 and AMOS 17.0.

### Common Method Bias Test

Since the study uses a single-source survey for data collection, there may be common method deviations. To control for this, we utilized the following methods. First, some remedial measures were taken during implementation of the questionnaire, such as ensuring the respondent’s anonymity, emphasizing that there is no right or wrong answer, and trying to reduce socially acceptable responses. Richman et al. (1999) believe that paper-pencil and electronic tests are less socially acceptable deviations than face-to-face interviews, especially when anonymous. Second, the Harman single factor method was used to test the common method deviation. Principal component analysis of all variables shows that the explanatory variance of the first factor before rotation is 12.648%, which is far less than the critical standard of 50%, indicating that the common method deviation of this study is within the acceptable range.

## Results

### Differences in Gender Role Characteristics Between Female and Male Entrepreneurs

The questionnaire used to collect data for this study contained a number of demographic variables, including age, education, type of company, time of establishment of the company, number of employees, and industry. When conducting statistical analysis, these variables were examined as potential control variables. However, since none of these characteristic variables showed significant differences in either gender role identification or ESE, they are not reported here.

Table 2 shows the tests of difference between the masculine and feminine gender role factors for male and female entrepreneurs. While male entrepreneurs were higher than female entrepreneurs in the masculine factors, and females were higher in the feminine factors, the only significant differences were in the factors “Forthright and Sincere” and “Friendly,” supporting Hypothesis 1.

**TABLE 2:** *T*-tests of gender role factors.

	Male (*N* = 265)		Female (*N* = 261)		*t*
		
	*Mean*	*SD*	*Mean*	*SD*	
**Masculine factors**					
Self-government	4.073	0.676	3.994	0.562	−1.45
Enterprise	2.811	0.911	2.810	0.828	0.01
Power	3.735	0.683	3.623	0.625	−1.95
Compete	3.874	0.633	3.805	0.604	−1.29
**Feminine factors**					
Forthright and sincere	2.384	0.868	2.977	0.704	8.60***
Friendly	3.762	0.696	4.001	0.539	4.39***
Empathy	3.839	0.694	3.944	0.609	1.84

***p < 0.001.

### Differences in ESE Between Female and Male Entrepreneurs at Entrepreneurship Stages

Table 3 shows the differences in the ESE of female and male entrepreneurs at the four stages of entrepreneurship. The results indicated no significant differences, supporting Hypothesis 2.

**TABLE 3:** *T*-tests of entrepreneurial self-efficacy in entrepreneurship stages.

ESE	Male		Female		*t*
		
	*Mean*	*SD*	*Mean*	*SD*	
Searching	3.709	0.862	3.778	0.873	0.90
Planning	3.376	0.963	3.456	0.905	0.99
Marshaling	3.348	0.825	3.402	0.866	0.74
Implementing	3.853	0.801	3.933	0.746	1.19

### The Relationship Between Gender Role Characteristics and ESE of Male and Female Entrepreneurs at Various Entrepreneurship Stages

Table 4 presents the correlations among the gender role factors and ESE at each of the entrepreneurship stages. Table 5 provides the findings of the regression analyses. The results show that for female entrepreneurs, the masculine factor of “Compete” and the feminine factor of “Friendly” were positively associated with ESE in the searching stage. For male entrepreneurs, the feminine factor of “Friendly” was positively associated with ESE in the searching, planning and marshaling stages, which partly supports Hypothesis 3.

**TABLE 4:** Correlations.

	Composite reliability	Convergence validity	Discrimination validity										
			
	CR	AVE	1	2	3	4	5	6	7	8	9	10	11
1. Self-government	0.812	0.465	**0.659**										
2. Enterprise	0.661	0.424	0.136**	**0.584**									
3. Power	0.708	0.447	0.329**	0.350**	**0.637**								
4. Compete	0.771	0.530	0.358**	0.332**	0.462**	**0.667**							
5. Forthright and sincere	0.682	0.462	−0.056	0.183**	0.055	0.017	**0.574**						
6. Friendly	0.604	0.380	0.163**	−0.011	0.179**	0.212**	0.236**	**0.589**					
7. Empathy	0.632	0.363	0.199**	0.059	0.220**	0.153**	0.234**	0.363**	**0.579**				
8. Searching stage	0.812	0.683	0.142**	0.084	0.169**	0.230**	0.022	0.224**	0.135**	**0.827**			
9. Planning stage	0.695	0.533	0.106*	0.102*	0.102*	0.150**	0.151**	0.211**	0.124**	0.614**	**0.730**		
10. Marshaling stage	0.772	0.461	0.095*	0.102*	0.056	0.132**	0.101*	0.118**	0.082	0.488**	0.557**	**0.679**	
11. Implementing stage	0.496	0.332	0.173	0.107*	0.153**	0.241**	0.031	0.089*	0.157**	0.411**	0.419**	0.490**	**0.576**

Factor reliability coefficients are provided in bold in the diagonal. *p < 0.05, **p < 0.01.

**TABLE 5:** Regression analysis.

		Entrepreneurial self-efficacy							

Gender role factor	Gender	ESE in searching stage		ESE in planning stage		ESE in marshaling stage		ESE in implementing stage	
					
		*Beta*	*t*	*Beta*	*t*	*Beta*	*t*	*Beta*	*t*
Self-government	Male	0.038	0.583	0.061	0.918	0.034	0.495	0.119	1.820
	Female	0.012	0.184	0.022	0.329	0.108	1.596	0.030	0.439
Enterprise	Male	−0.003	−0.041	0.007	0.107	0.166	2.382*	0.070	1.040
	Female	0.067	0.966	0.112	1.626	−0.017	0.239	−0.041	−0.598
Power	Male	0.125	1.843	0.007	0.102	−0.065	−0.911	−0.033	−0.481
	Female	−0.051	−0.663	−0.058	−0.754	−0.015	−0.192	0.106	1.376
Compete	Male	0.123	1.720	0.049	0.669	0.041	0.543	0.239	3.291***
	Female	0.187	2.574*	0.130	1.794	0.115	1.575	0.122	1.679
Forthright and sincere	Male	0.101	1.527	0.043	0.628	−0.040	−0.578	0.104	1.546
	Female	−0.029	−0.433	−0.001	−0.010	0.090	1.349	0.099	1.491
Friendly	Male	0.172	2.613**	0.159	2.359*	0.138	2.005*	−0.050	−0.749
	Female	0.171	2.545*	0.168	2.499*	0.006	0.094	0.018	0.264
Empathy	Male	−0.084	−1.329	0.145	2.220*	0.005	0.074	−0.032	−0.504
	Female	−0.006	−0.096	0.052	0.817	0.124	1.957	−0.002	−0.024
F	Male	5.889		3.612		1.994		4.980	
	Female	2.585		2.418		2.247		2.183	
R^2^	Male	0.115		0.065		0.026		0.095	
	Female	0.067		0.037		0.032		0.031	

*p < 0.05, **p < 0.01, ***p < 0.001. Male: n = 265; Female: n = 261.

The “Friendly” factor was positively associated with ESE in the planning stage for female entrepreneurs. The feminine factor “Empathy” was positively associated with male entrepreneurs’ ESE in the planning stage as well. The “Enterprise” and “Compete” factors were positively associated with male entrepreneurs’ ESE in the marshaling and implementing stages respectively, which supported Hypothesis 4.

Overall, the “Compete” and “Friendly” factors appeared to be more frequently associated with ESE than other factors. In addition to the typically masculine gender role characteristics of “Compete” and “Enterprise,” the feminine gender role characteristics of “Friendly” and “Empathy” appear to be important for male entrepreneurs’ ESE in multiple entrepreneurship stages. Likewise, in addition to the typically feminine gender role characteristic of ‘Friendly,” the masculine gender role characteristic of “Compete” appears to be important for female entrepreneurs’ ESE in the searching stage of entrepreneurship.

## Discussion

In this study, we sought to investigate the differences in and relationship between the gender role characteristics and ESE of 261 female and 256 male entrepreneurs. The results reveal that the only significant mean differences between female and male entrepreneurs occur for the feminine gender role factors of “Forthright and Sincere” and “Friendly,” supporting Hypothesis 1. This result is compatible with previous research findings (Williams and Best, 1982; Stephen and Mary, 2008; Zhi, 2011) indicating that men (including men exhibiting feminine characteristics) and women exhibiting masculine traits demonstrate the psychological characteristics of self-concept and self-esteem typically associated with suitability for leadership (Kent and Moss, 1994; McCabe et al., 2006). However, the results also showed that female entrepreneurs exhibit higher feminine gender role factors than male entrepreneurs, suggesting that traditionally feminine gender role characteristics are still important for female entrepreneurs to display.

Previous research results are mixed on the influence of gender on ESE, which may be summarized as either women’s ESE is lower than men’s (Scherer et al., 1990; Wilson et al., 2007) or there is no gender difference in ESE (Zhao et al., 2005; Stephen and Mary, 2008). However, these studies primarily utilized samples of MBA students and non-entrepreneurs. In the present study which investigated established entrepreneurs, who may be expected to have higher ESE than non-entrepreneurs, we found no gender differences in ESE exhibited across four different entrepreneurship stages, a result that is compatible with Chen et al. (1998). It may be that in today’s China, although women share equal rights and social status as men, long-term gender stereotypes may still prevail. For women entrepreneurs to succeed, therefore, they may need to exhibit the same levels of entrepreneurial intention and self-efficacy as their male counterparts do. Women also tend to have a higher drive to succeed and persist in business given the opportunity offers it for work-family balance (Baron and Henry, 2011).

In terms of the tests of association between gender role factors and ESE at various entrepreneurship stages, the results show that the feminine factor “Friendly” had a positive influence on male and female entrepreneurs’ ESE at the searching stage, partially supporting Hypothesis 3. Within the structure of gender role orientation, “Friendly” is defined as considerate, understanding and peaceful, which allows people to more easily establish positive interpersonal relationships with others, which is essential to obtaining information and discovering opportunities during the entrepreneurial process. For female entrepreneurs, in addition to “Friendly,” the masculine characteristic of “Compete” also had a positive impact on their ESE in the searching stage. While not in accordance with Hypothesis 3, we may infer that female entrepreneurs having the masculine characteristic of “Compete” together with the feminine factor of “Friendly” likely see themselves as confident, strong in action and leadership, ambitious, resourceful, and having ease in establishing good interpersonal relationships with others, all of which are more conducive to gaining new business opportunities.

The study also found that masculine gender role factors are not associated with male and female entrepreneurs’ ESE in the planning stage of entrepreneurship. However, the feminine factor “Friendly” appears to play an active role in this stage as well, for both male and female entrepreneurs. In planning the birth of an enterprise, entrepreneurs may be confronted with uncertainties, such as changes in the external environment, uncertainties about partners, etc. Thus, entrepreneurs must be adaptive, nimble and flexible in this stage. When facing uncertainties, “Friendly” enables male and female entrepreneurs to adapt to possible changes through communication and connection with others. Additionally, the feminine factor “Empathy” was positively associated with male entrepreneurs’ ESE in the planning stage. The factor “Empathy” was defined as compassionate, cheerful, and affectionate in the present study, representing an understanding of the perspectives and constraints of others. In the planning stage, where it would be important to coordinate with others, empathy and emotional intelligence are important. Previous research has shown that emotional intelligence has a positive influence on ESE (Leonidas et al., 2009). The results indicate that empathy is particularly important for male entrepreneurs’ ESE in the planning stage, possibly because it may help them to understand, connect, and relate with others.

The results also indicated that for male entrepreneurs, the masculine factors “Enterprise” and “Compete” were positively associated, respectively, with their ESE at the marshaling and the implementing stage, and the feminine factor “Friendly” was positively associated with their ESE at the marshaling stage. For female entrepreneurs, neither masculine nor feminine factors were associated with their ESE at the marshaling and the implementing stages, which supports Hypothesis 4. According to Stephen and Mary (2008), during the marshaling and implementing stages of entrepreneurship, typical masculine teams show a higher level of ESE than typical female teams. “Enterprise” and “Compete” were defined as confident, aggressive, dominant, and spearheading in the present study, which are typically masculine characteristics. During the entrepreneurial process, the searching and the planning stages mainly cover the preparation of business while not requiring as strong a demand of leadership to spearhead, coordinate, and control others. The marshaling and the implementing stages, on the other hand, require entrepreneurs to demonstrate their leadership skills, self-confidence, and power to influence investors, suppliers, and new employees, all of which are typical masculine factors. It is interesting that these associations were obtained only for male entrepreneurs; it seems that male but not female entrepreneurs’ ESE is bolstered by these masculine factors in the marshaling and implementing stages. Additionally, at the marshaling stage, entrepreneurs need excellent communication skills to persuade and encourage others, so the factor “friendly” is important for male entrepreneurs.

### Contributions and Implications

The present study’s theoretical contributions are as follows. First, we studied a novel phenomenon in the literature on ESE. Most previous studies have employed ESE as an antecedent variable to explore its influence on entrepreneurial intent and entrepreneurial performance. In this study, we employed ESE as an outcome variable to explore the influence of entrepreneurs’ gender role on ESE in various stages of entrepreneurship and between male and female entrepreneurs. Our study enriches the extant ESE knowledge base in these respects.

Second, the integration of the Bem Sex Role Inventory (BSRI) in the present study provides new and nuanced insights about the influence of gender roles in the development of ESE. Our study explores the content and structure of the BSRI, dividing gender roles into seven factors, of which three are feminine and four are masculine, comparing and analyzing the differences between female and male entrepreneurs’ on these gender role factors and ESE. Previous studies using BSRI have divided gender roles into only two dimensions of masculinity and femininity. Thus, this study not only reveals the influence of gender role identification on ESE, but also provides a more nuanced perspective on gender roles. Third, our data were collected from practicing entrepreneurs, which is another strength of this study. Unlike previous studies, which mostly focused on MBA students, our study focused on male and female entrepreneurs, ensuring that the ESE being captured was closer to reality than aspiration.

There are two practical contributions of this study. First, it eliminates the stereotype that men are more suitable for entrepreneurship than women, and provides strong evidence for female entrepreneurship management. In recent years, although women have become more active in entrepreneurship and the trend of female entrepreneurship has increased annually, men are still generally considered to be more suitable for entrepreneurship than women. By comparing 261 female entrepreneurs and 265 male entrepreneurs, our study found that there is no difference between female entrepreneurs and male entrepreneurs in terms of their ESE. In practice, thus, society should encourage and attach equal importance and support to female entrepreneurship as to male entrepreneurship, providing women entrepreneurs with a larger space and platform than they currently have, so that they can give full play to their talents and achieve entrepreneurial performance.

Second, our results suggest that both masculinity and femininity are important for entrepreneurship. The present study found that male entrepreneurs and female entrepreneurs have no differences in masculine characteristics; that is, female entrepreneurs also have masculine characteristics, indicating that masculine characteristics are essential for entrepreneurship. At the same time, the feminine intuitive factor played an important role in opportunity identification in the search stage, and the feminine factor of friendly was important in multiple stages. In practice, we should not only pay attention to the cultivation of the male characteristics of female entrepreneurs, but also cultivate the female characteristics of male entrepreneurs, so as to make entrepreneurs more effective in all stages of the entrepreneurial life cycle.

### Limitations and Future Research

While the present research verified the hypotheses, there are certain limitations, the first of which is the limited representativeness of the sample. Respondents were drawn from the developed regions of East China, where the entrepreneurial environment is rich enough to drive strong entrepreneurial motivations. Likely our respondents are opportunity-oriented entrepreneurs, and thus the results of this study may not represent entrepreneurship in other regions in China or in other countries. Second, although we launched remedial measures to control for the use of cross-sectional data, common method bias is still an issue. In this regard, the questionnaire deployed was anonymous and we emphasized to respondents that there are no right or wrong answers. Additionally, we intentionally avoided questions having social desirability so as to further lower common method biases. Richman et al. (1999) suggested that pen-and-paper tests and electronic surveys show less social desirability bias than face-to-face interview, especially when responses remain anonymous. We also conducted tests to ensure that common method bias was within the acceptable range. To overcome these limitations, future research should undertake cross-cultural comparisons of gender role characteristics and ESE, and consider more qualitative explorations to tease out the nuances of gender role orientations and entrepreneurial self-efficacy displayed by female and male entrepreneurs.

## Data Availability Statement

The raw data supporting the conclusions of this article will be made available by the authors, without undue reservation.

## Ethics Statement

The studies involving human participants were reviewed and approved by the Human Participants Ethics Committee of Shanghai Normal University, China. The patients/participants provided their written informed consent to participate in this study. Written informed consent was obtained from the individual(s) for the publication of any potentially identifiable images or data included in this article.

## Author Contributions

CL, DB, and XG have been engaged in research on organizational behavior. They have done many researches on organizational culture and female entrepreneurship and have published dozens of research manuscripts in recent years. CL and XG has presided over two National Social Science fund project, three provincial-level projects, and many other projects. CL’s monograph Organizational Culture: from the Perspective of Organizational Effectiveness won the second prize of the 12th Philosophy and Social Science Outstanding Achievements of Shanghai (China) in 2014. All authors contributed to the article and approved the submitted version.

## Conflict of Interest

The authors declare that the research was conducted in the absence of any commercial or financial relationships that could be construed as a potential conflict of interest.
