# Supplementary material for: Gender Role Characteristics and Entrepreneurial Self-Efficacy: A Comparative Study of Female and Male Entrepreneurs in China
Source: Front Psychol. 2020 Dec 17;11:585803. doi: 10.3389/fpsyg.2020.585803 (PMC7773753; doi:10.3389/fpsyg.2020.585803)
Supplement: Supplementary file 1 [file Data_Sheet_1.pdf]

Appendix 2. Gender Role Factor Analysis: EFA Results

Rotated Component Matrix[a]

		Component						
		1	2	3	4	5	6	7
Self-government	Self-made	.776						
	Autocratic	.765						
	Strong-minded	.724						
	Independent	.672						
	Be willing to declare	.655						
Forthright and Sincere	Compassionate		.785					
	Cheerful		.767					
	Affectionate		.634				.357	
	Child-friendly		.517			.290	-.318	-.320
Friendly	Childish			.752				
	Tender			.751				
	Sympathetic		.476	.575				
	Understanding			.565				.346
Enterprise	Ambitious				.807			
	Aggressive				.715			
	Positive				.552			
Power	Dominant					.676		
	As a leader					.630		
	Leadership skill	.301				.563		.321
Empathy	Child-like						.738	
	Aspire to placate the Hurt feelings						.637	
	Delicate			.336			.631	
Compete	Kinetic							.740
	Competitive				.322			.632
	Analytical					.313		.532

[a.] Extraction Method: Principal Component Analysis. Rotation Method: Varimax with Kaiser Normalization. Rotation converged in 7 iterations. Small coefficients >.03 suppressed.
