# Supplementary material for: Gender Role Characteristics and Entrepreneurial Self-Efficacy: A Comparative Study of Female and Male Entrepreneurs in China
Source: Front Psychol. 2020 Dec 17;11:585803. doi: 10.3389/fpsyg.2020.585803 (PMC7773753; doi:10.3389/fpsyg.2020.585803)
Supplement: Supplementary file 2 [file Table_1.pdf]

Appendix 1: Questionnaire

Dear Madam/ Sir,

Thank you for participating in this survey! The purpose of this survey is to understand the characteristics and entrepreneurial situation of male/female entrepreneurs in various regions of the country. There is no right or wrong option in this questionnaire, please fill it out according to the actual situation of your company. The results of this survey are only for research and not for other purposes. We will keep the information you filled in confidential. It will take you about 10 minutes to fill out the questionnaire. Thank you for your support!

Part One: Basic Information

1. Age：① 20-30 years old；② 31-40 years old； ③ 41-50 years old；④ above 51 years old
2. Education：① High school and below；② community College； ③ Bachelor degree；
   ④Master degree and above
3. Enterprise nature：①Family business；②General private business；③ Self-employed business
4. Enterprise creation time：① Below 1 year；② 1-3 years； ③ 4-5 years；④ 6-10 years；
   ⑤above 11 years
5. No. of employees：① 1-10；② 11-50； ③ 51-99；④100-499；⑤Above 500
6. Industry：① Manufactory；② Service/Trade； ③High technology；
   ④Finance/Real Estate；⑤Other

Part Two: BSRI

Explanation: For the following descriptions, please select the degree to which you meet or do not meet these descriptions (1=very inconsistent; 2=Comparatively inconsistent; 3=Indifferent to inconsistent or inconsistent; 4=Comparatively in line; 5=very in line

1、Affectionate	1	2	3	4	5
2、Cheerful	1	2	3	4	5
3、Innocent	1	2	3	4	5
4、Compassionate	1	2	3	4	5

5、Don't use stern language	1	2	3	4	5
6、Be eager to the console the hurt feelings	1	2	3	4	5
7、Feminine	1	2	3	4	5
8、Flattering	1	2	3	4	5
9、Moderate	1	2	3	4	5
10、Credulous	1	2	3	4	5
11、Love children	1	2	3	4	5
12、Loyal	1	2	3	4	5
13、Be sensitive to the needs of others	1	2	3	4	5
14、Bashful	1	2	3	4	5
15、Tender	1	2	3	4	5
16、Sympathetic	1	2	3	4	5
17、Naïve	1	2	3	4	5
18、Reasonable	1	2	3	4	5
19、Passionate	1	2	3	4	5
20、Yielding	1	2	3	4	5
21、As a leader	1	2	3	4	5
22、Aggressive	1	2	3	4	5
23、Ambitious	1	2	3	4	5
24、Analytic	1	2	3	4	5
25、Arbitrary	1	2	3	4	5
26、Moveable	1	2	3	4	5
27、Competitive	1	2	3	4	5
28、Maintain confidence	1	2	3	4	5
29、Dominant	1	2	3	4	5
30、Powerful	1	2	3	4	5
31、Capable of leadership	1	2	3	4	5
32、Independent	1	2	3	4	5
33、Individualistic	1	2	3	4	5
34、Decisive	1	2	3	4	5
35、Masculine	1	2	3	4	5
36、Self-reliance	1	2	3	4	5
37、Autocratic	1	2	3	4	5
38、Strong-minded	1	2	3	4	5
39、Be willing to declare	1	2	3	4	5
40、Adventurous	1	2	3	4	5

Part Three: ESE

Explanation: For the following descriptions, please select the degree to which you meet or do not meet these descriptions (1=very inconsistent; 2=Comparatively

inconsistent; 3=Indifferent to inconsistent or inconsistent; 4=Comparatively in line; 5=very in line

1、Conceive a unique idea for a business	1	2	3	4	5
2、Identify market opportunities for a new business	1	2	3	4	5
3、Plan a new business	1	2	3	4	5
4、Write a formal business plan	1	2	3	4	5
5、Raise money to start a business	1	2	3	4	5
6、Convince others to invest in your business	1	2	3	4	5
7、Convince a bank to lend you money to start a business	1	2	3	4	5
8、Convince others to work for you in your new business	1	2	3	4	5
9、Manage a small business	1	2	3	4	5
10、Grow a successful business	1	2	3	4	5
